# Charge Carrier Regulation for Efficient Blue Quantum-Dot Light-Emitting Diodes Via a High-Mobility Coplanar Cyclopentane[*b*]thiopyran Derivative

**DOI:** 10.1021/acs.nanolett.4c00883

**Published:** 2024-04-16

**Authors:** Fensha Cai, Hao Zong, Meng Li, Chenguang Li, Guangguang Huang, Jorge Pascual, Chao Liang, Zhenhuang Su, Zhe Li, Xingyu Gao, Bo Hou, Shujie Wang, Gang Zhou, Zuliang Du

**Affiliations:** † Key Lab for Special Functional Materials of Ministry of Education, National & Local Joint Engineering Research Center for High-efficiency Display and Lighting Technology, School of Materials Science and Engineering, and Collaborative Innovation Center of Nano Functional Materials and Applications, Henan University, Kaifeng 475004, P. R. China; ‡ Lab of Advanced Materials, State Key Laboratory of Molecular Engineering of Polymers, Fudan University, Shanghai 200438, P. R. China; § Polymat, University of the Basque Country UPV/EHU, Donostia-San Sebastian 20018, Spain; ∥ MOE Key Laboratory for Nonequilibrium Synthesis and Modulation of Condensed Matter, School of Physics, Xi’an Jiaotong University, Xi’an 710049, P. R. China; ⊥ Shanghai Synchrotron Radiation Facility (SSRF), Shanghai Advanced Research Institute, Chinese Academy of Sciences, 239 Zhangheng Road, Shanghai 201204, P. R. China; # School of Engineering and Materials Science (SEMS), Queen Mary University of London, London E1 4NS, United Kingdom; ∇ School of Physics and Astronomy, Cardiff University, Cardiff CF24 3AA, Wales, United Kingdom

**Keywords:** blue quantum dot light-emitting diodes, charge balance, hole transport layer, high hole mobility, cyclopentane[*b*]thiopyran derivative

## Abstract

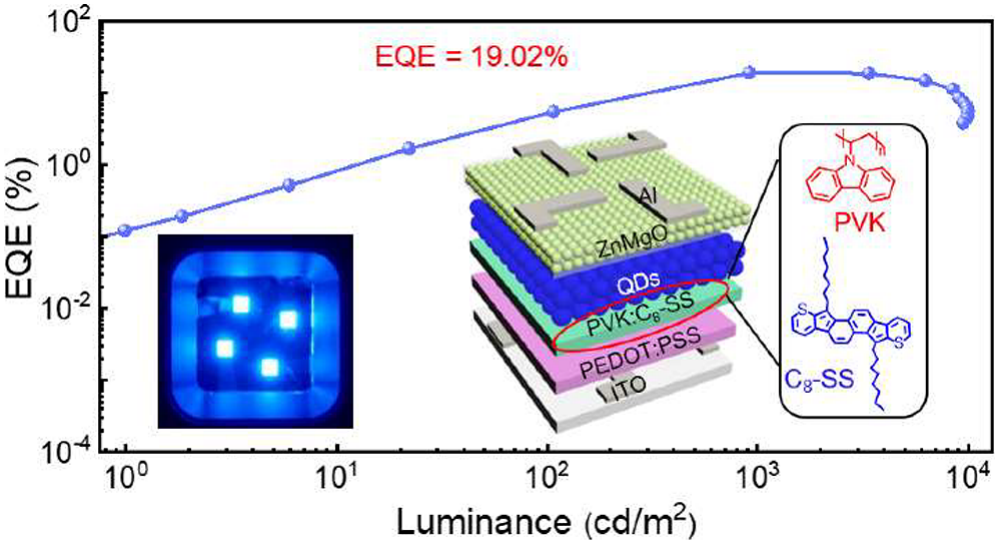

The performance of blue quantum dot light-emitting diodes (QLEDs) is limited by unbalanced charge injection, resulting from insufficient holes caused by low mobility or significant energy barriers. Here, we introduce an angular-shaped heteroarene based on cyclopentane[*b*]thiopyran (C_8_–SS) to modify the hole transport layer poly-*N*-vinylcarbazole (PVK), in blue QLEDs. C_8_–SS exhibits high hole mobility and conductivity due to the π···π and S···π interactions. Introducing C_8_–SS to PVK significantly enhanced hole mobility, increasing it by 2 orders of magnitude from 2.44 × 10^–6^ to 1.73 × 10^–4^ cm^2^ V^–1^ s^–1^. Benefiting from high mobility and conductivity, PVK:C_8_–SS-based QLEDs exhibit a low turn-on voltage (*V*_on_) of 3.2 V. More importantly, the optimized QLEDs achieve a high peak power efficiency (PE) of 7.13 lm/W, which is 2.65 times that of the control QLEDs. The as-proposed interface engineering provides a novel and effective strategy for achieving high-performance blue QLEDs in low-energy consumption lighting applications.

Colloidal semiconductor quantum dots (QDs) have rapidly advanced the field of light-emitting devices due to high photoluminescence quantum yield (PLQY), bandgap-tunability, high color purity, and high photochemical stability.^1−3^ These characteristics make QD-based light-emitting diodes (QLEDs) promising candidates for display and lighting technologies. Tremendous progress has recently been achieved in red and green QLEDs, with external quantum efficiency (EQE) over 20%.^4,5^ The operational lifetimes of red and green QLEDs have achieved T_95_ (@1000 cd/m^2^) values of 48,000 and 7200 h, respectively,^5,6^ meeting commercial requirements. However, it remains a significant challenge to develop high-performance blue QLEDs.

State-of-the-art QLEDs primarily adopt zinc oxide nanoparticles (ZnO NPs) as electron transport layer (ETL) due to their high electron mobility and suitable interface electronic landscape with QDs.^7−9^ Poly-*N*-vinylcarbazole (PVK) is commonly used as hole transport layers (HTLs) due to its deep highest occupied molecular orbital (HOMO).^10,11^ However, the low hole mobility of PVK causes insufficient hole injection into the QDs, inducing a charge imbalance. This will increase the Auger recombination and severely limit the device performance.^12,13^ Several approaches have been developed to enhance hole injection rates, such as designing new hole transport materials (HTMs),^14,15^ utilizing double-layer HTLs,^16,17^ and modifying the HTL with organic molecules.^18−22^ Although these efforts have markedly enhanced the device performance, the efficiency of blue QLEDs is still inferior to that of other QLEDs. In addition, organic molecules such as CBP,^19^ Li-TFSI,^20^ or TAPC^21^ resulted in aggregation and inhomogeneous films, which limit the improvement of device performance. Therefore, developing new organic molecules to simultaneously enhance the PVK mobility and form homogeneous films is of great importance.

Cyclopentane[*b*]thiopyran has become one of the most important semiconductors in optoelectronic devices due to its unique charge transport characteristics.^23,24^ In our previous work, we reported the straightforward synthesis of a series of angular-shaped heteroarenes based on cyclopentane[*b*]thiopyran, that is, C_n_-SS (n = 4, 6, 8, 10), with different linear alkyl groups.^25^ Among them, C_8_–SS exhibits high mobility up to 1.1 cm^2^ V^–1^ s^–1^ due to the coplanar conjugated π···π and S···π interactions. Therefore, we employed C_8_–SS to modify the PVK HTL in blue QLEDs. We revealed that the hole mobility of PVK:C_8_–SS showed a 2 orders of magnitude improvement and greatly increased film uniformity. Compared with the control device, the modified device exhibits a low turn-on voltage (*V*_on_) of 3.2 V; we explained the mechanism and demonstrated this point by capacitance–voltage (*C–V*) and transient electroluminescence (TrEL) measurements. Accordingly, the QLEDs with PVK:C_8_–SS HTL exhibited an EQE of 19.02% and a peak power efficiency (PE) of 7.31 lm/W cd/m^2^, which are 1.78 and 2.65 times those of the control devices. Remarkably, a low *V*_on_ (defined as the voltage at which the luminance is 1 cd m^–2^) of 3.2 V makes important progress for using blue QLEDs as a backlight in display and ambient lighting technologies with a lower driving voltage and less energy consumption.

To investigate the film quality after introducing C_8_–SS into PVK, we carried out atomic force microscopy (AFM) to study the surface topography of PVK and PVK:C_8_–SS (Figure 1). The molecular structures of PVK and C_8_–SS are shown in Figure S1. The PVK:C_8_–SS film exhibited a smaller root-mean-square (RMS) (0.316 nm) than PVK (0.540 nm), indicating that C_8_–SS homogenizes the PVK film surface, which contributes to reducing the leakage current in QLEDs.^26^Figure 1c presents a height profile plot of PVK and PVK:C_8_–SS, where the smaller RMS suggests higher film quality. We performed conductive AFM (c-AFM) to characterize the local charge transport on a microscale (Figures 1d,e).^27^ The vertical current of the PVK:C_8_–SS film was found to be two times higher than that of PVK, with a more uniform current distribution (Figure 1f), indicating C_8_–SS will improve PVK film’s conductivity. This can be attributed to several factors. First, the calculated transfer integral of single-crystal C_8_–SS is 26 meV (Figure S2). This suggests that the frontier orbitals overlap, facilitating hole transport among adjacent slipped-stacking molecules of C_8_–SS.^28^ Moreover, when C_8_–SS is introduced into PVK, the intermolecular S···π and S···S interactions in the PVK:C_8_–SS result in a significant overlap of HOMOs between neighboring molecules. Consequently, the incorporation of S atoms enhances intramolecular charge transfer interactions, leading to increased intermolecular hole transport mobilities.^29^Figure 1g–i shows the 2D grazing incidence X-ray diffraction (GIXRD) patterns of QDs on PVK and PVK:C_8_–SS substrates. Both samples present a uniform diffraction ring, indicating a consistent crystalline orientation of the films.^12^ The scattering ring in the PVK:C_8_–SS/QDs film appears considerably sharper compared to that in the PVK-based sample, indicating that introducing C_8_–SS into PVK increases the QDs’s coverage per unit area, thus enhancing the diffraction intensity.^12,30^ The plots of the azimuthally (90°) integrated intensities in both samples revealed this feature more clearly, as shown in Figure 1i. The peaks at *q* = 19.53 nm^–1^ for PVK:C_8_–SS/QDs are stronger than those for the QDs on the PVK substrate. The enhanced diffraction intensity confirmed the improved performance of the QD film.

**Figure 1:**
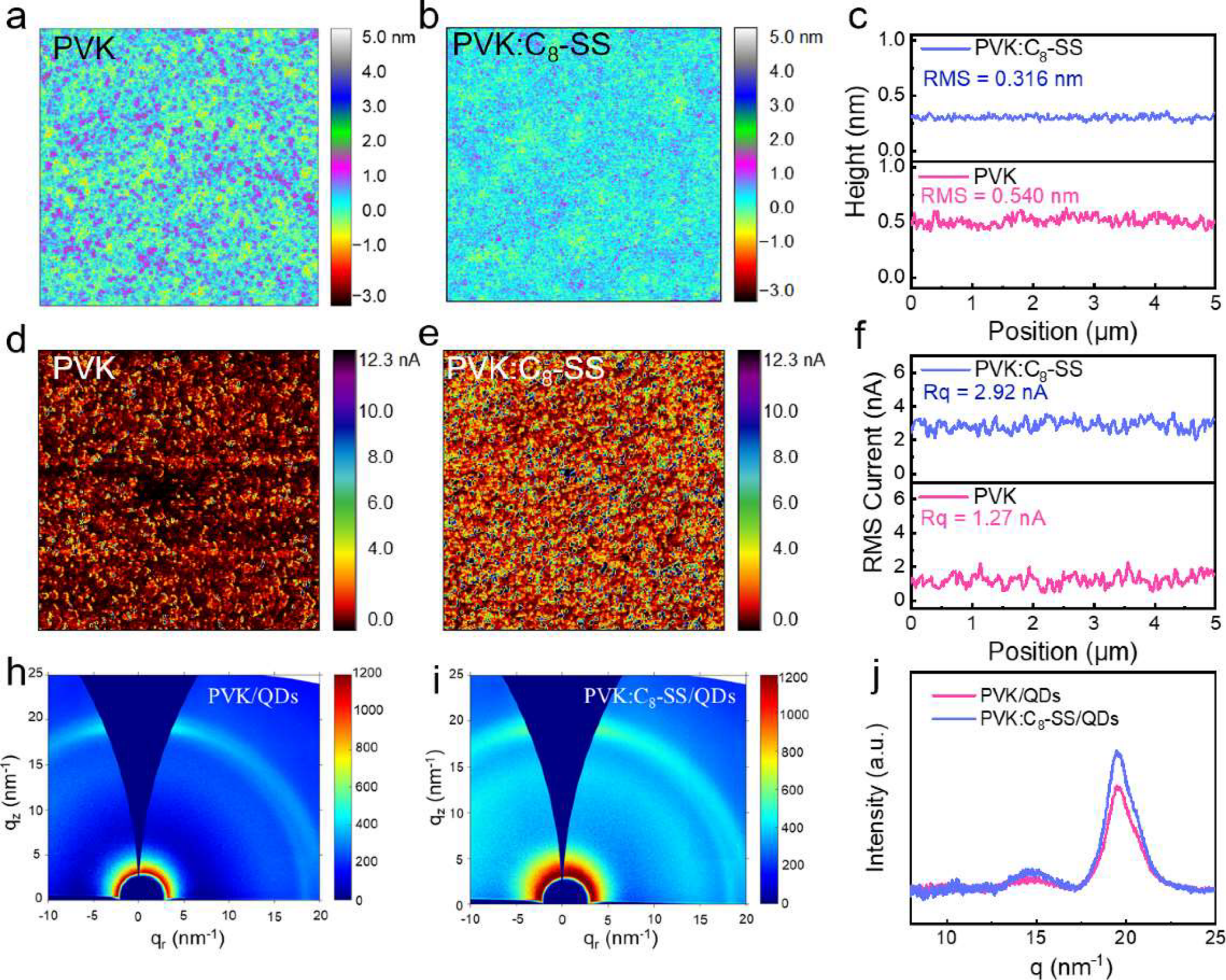
AFM images of (a) PVK and (b) PVK:C_8_–SS. (c) RMS of the height profile plot for PVK and PVK:C_8_–SS. Conductive-AFM images at −2 V bias of (d) PVK and (e) PVK:C_8_–SS. (f) Rq of profile plot for PVK and PVK:C_8_–SS (All image sizes are 5 × 5 μm). 2D GIXRD patterns of the QD films on (g) PVK and (h) PVK:C_8_–SS substrates. (i) Azimuthally integrated intensity plots for the surface 2D GIXRD patterns along the direction of the outside surface for QDs on PVK and PVK:C_8_–SS HTLs.

The hole mobility of PVK and PVK:C_8_–SS films was determined using the space charge-limited current (SCLC) method (Figure 2a,b).^31−33^ The device structure is ITO/PEDOT:PSS/HTLs/MoO_3_/Al. The hole mobility (μ) is extracted by fitting the *J–V* curves using the Mott–Gurney law
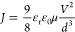
1where *J* represents the current density, ε_r_ is the relative dielectric constant, ε_0_ is the vacuum dielectric constant, and *d* is the thickness of the HTL layer. The calculated hole mobility of PVK:C_8_–SS is 1.73 × 10^–4^ cm^2^ V^–1^ s^–1^, which is 2 orders of magnitude larger than that of PVK (2.44 × 10^–6^ cm^2^ V^–1^ s^–1^). Notably, high hole mobility promotes hole transfer and injection into QDs, promoting charge balance. We evaluated the impact of interface modification on carrier dynamics by time-resolved PL (TRPL) for the pristine QDs, PVK/QDs, and PVK:C_8_–SS/QDs films (Figure 2c). The TRPL curves were fitted by a biexponential decay model, and the results are presented in Table S1. The average exciton lifetimes (τ_ave_) decreased from 6.63 ns (pristine QDs) to 4.13 ns (PVK/QDs) and increased to 5.40 ns for PVK:C_8_–SS/QDs. We attribute this to suppressing the fluorescence quenching originating from charge transfer from QDs to the PVK HTL.^5,34^ We calculated the charge-transfer rate (*k*_CT_) and efficiency (η_CT_) of the charge carrier from QDs to HTL using the following equations^35^
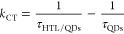
2
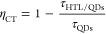
3where τ_QDs_ and τ_HTL/QDs_ represent the average lifetimes of QDs and HTL/QDs samples, respectively. For PVK/QDs, *k*_CT_ and η_CT_ are 9.13 × 10^7^ s^–1^ and 37.71%, respectively. For PVK:C_8_–SS/QDs, *k*_CT_ and η_CT_ notably decreased to 3.44 × 10^7^ s^–1^ and 18.55%, respectively, indicating that C_8_–SS can effectively suppress the charge-transfer process. Steady-state PL further confirmed this result (Figure S3). The enhanced PL intensity of the PVK:C_8_–SS compared to the PVK film is due to reducing exciton quenching of QDs.

**Figure 2:**
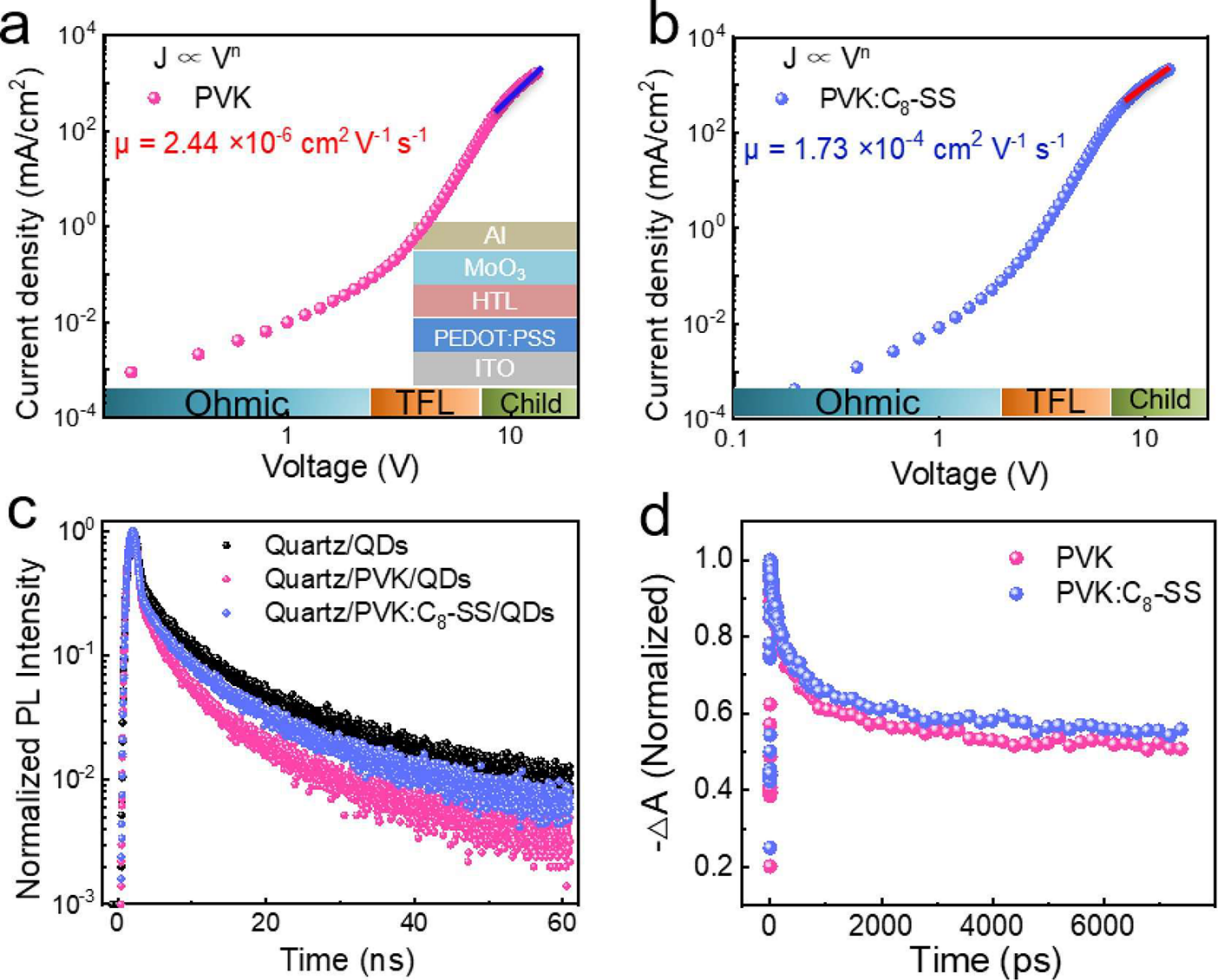
Hole mobility for (a) PVK and (b) PVK:C_8_–SS using the SCLC model. (inset) The device structure of ITO/PEDOT:PSS/HTLs/MoO_3_/Al. TFL stands for trap-filled limit. (c) TRPL decay for the pristine QDs, PVK/QDs, and PVK:C_8_–SS/QDs films deposited on quartz substrates. (d) TA delay of PVK/QDs and PVK:C_8_–SS/QDs films deposited on quartz substrates at 454 nm.

We performed additional transient absorption (TA) spectroscopy measurements to gain further insight into this mechanism. Figure S4 displays the TA response of the QD films on different HTLs after excitation (365 nm). The ground-state bleaching maximum is approximately 450 nm, consistent with the exciton absorption position in the UV–vis absorption spectrum (Figure S5). Figure 2d compares the transition dynamics at a wavelength of 454 nm for the two samples. The C_8_–SS-treated sample exhibits a longer exciton lifetime than that of the control sample, indicating suppressed electron transfer.^36−38^

To demonstrate the advantage of modifying PVK with C_8_–SS, blue QLEDs were fabricated with the architecture of ITO/PEDOT:PSS/PVK:C_8_–SS/QDs/ZnMgO/Al (Figure 3a). Figure 3b shows an energy-level diagram. The CdSe/ZnSe/ZnS core/shell QDs are the same as ours reported previously, with a PLQY of approximately 65% and an emission wavelength of 459 nm.^39^ Different amounts of C_8_–SS were explored, and the devices containing 3.6 wt % showed superior performance (Figure S6). The emission peak of the QLEDs was located at 464 nm, and the EL spectra exhibit no obvious redshift under different biases (Figure 3c), demonstrating excellent EL spectral stability. The inset in Figure 3c displays photos of QLEDs operating at 3.5 V, showing color-saturated and uniform emission. The Commission Internationale de I’Eclairage color coordinates were (0.1443, 0.0786), indicating highly saturated emission colors (Figure S6).^40^Figure 3d,e shows the current density–voltage-luminance (*J-V-L*), EQE-luminance, and power efficiency-luminance (PE-*L*) characteristics of the devices. The PVK:C_8_–SS-QLEDs exhibited a peak EQE of 19.02% and a peak PE of 7.31 lm/W, which were enhanced by 78% and 165% compared with PVK devices (10.71% and 2.76 lm/W). Detailed device parameters are summarized in Table S2. We attribute the enhanced device performance to the extremely high hole mobility of PVK:C_8_–SS and the considerably reduced leakage current. High hole mobility facilitates hole injection into the QDs, achieving charge balance and reducing Auger recombination. Additionally, PVK:C_8_–SS can suppress electron leakage toward the HTL, restraining nonradiative recombination.^42^ Importantly, the *V*_on_ dropped from 5.0 V (control device) to 3.2 V for PVK:C_8_–SS-based QLEDs, and this result is particularly promising, considering the low-energy consumption requirements in practical applications.

**Figure 3:**
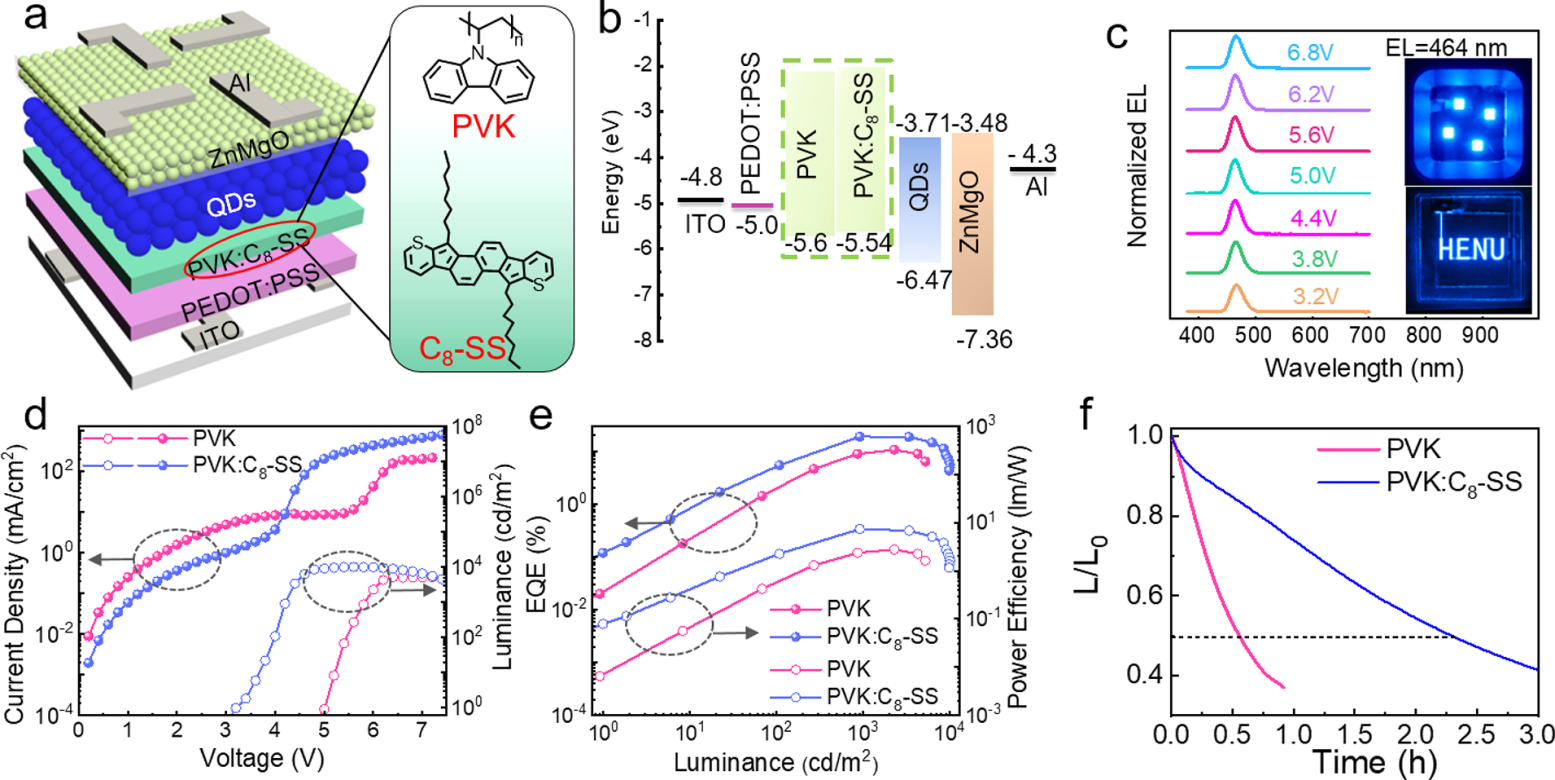
Device structure and performance. (a) Device structure. (b) Energy-level diagram. (c) EL spectra. (inset) Photographs of devices at 3.5 V. (d) *J-V-L* characteristics. (e) EQE-L-PE. (f) Operational lifetime.

To study why introducing 3.6 wt % C_8_–SS outperformed, we conducted ultraviolet photoelectron spectroscopy (UPS) to investigate the energy levels of PVK and PVK:C_8_–SS films (Figure S8). The HOMO energy level of PVK is 5.6 eV, slightly elevated to 5.54 eV for 3.6 wt % C_8_–SS, but upshifts to 5.47 eV for 6.0 wt % C_8_–SS. These results indicate that adding excess C_8_–SS introduces an increased hole injection barrier, which hinders hole injection. Additionally, adding excess C_8_–SS reduces the transmittance of PVK films (Figure S9), which is detrimental to the device performance because of a reduction in optical coupling efficiency.^41^ The average EQE from 12 PVK:C_8_–SS-based devices reached 17.43% (Figure S10), indicating excellent reproducibility.

Next, we investigated the environmental stability of PVK and PVK:C_8_–SS films and their device lifetime. Under an optical microscope (Figure S11), abundant cracks were observed on the PVK film, and these cracks become more pronounced after 5 h. However, the PVK:C_8_–SS film exhibits a more uniform and smoother surface, with no obvious change after aging, indicating higher stability. The contact angles increased remarkably after PVK was treated with C_8_–SS, indicating enhanced hydrophobic character due to the hydrophobic alkyl chains (Figure S12). The operational lifetime is shown in Figure 3f. The T_50_ lifetime of the modified device reached 2.3 h at a constant current of 38 mA cm^–2^, corresponding to an initial luminance of 3208 cd m^–2^. The measure T_50_ of the control device was 0.55 h at an initial luminance of 1714 cd m^–2^. The device lifetime (T_50_@100 cd m^–2^) was extended from 92 to 1183 h, achieving a remarkable 12-fold improvement through accelerated aging and the utilization of formula conversion.^43^ The enhanced lifetime was attributed to effective hole injection, decreasing Auger recombination, and electron leakage.

To further explain the improvement in device performance, we fabricated a hole-only device (HOD) with the structure of ITO/PEDOT:PSS/PVK or (PVK:C_8_–SS)/QDs/MoO_3_/Al and an electron-only device (EOD) with the structure of ITO/ZnMgO/QDs/ZnMgO/Al (Figure S13). The current density of PVK:C_8_–SS-based HOD is markedly increased by approximately 2 orders magnitude compared with the PVK HOD. The smaller difference in the current density of HOD and EOD indicates that C_8_–SS-modified PVK can balance carriers, contributing to achieving efficient EL. Moreover, the *C–V* characteristics of the QLEDs were investigated to analyze the charge injection (Figure 4a).^44^ The PVK:C_8_–SS-QLEDs show a voltage of 3.4 V at the peak capacitance that is smaller than that of the control devices (5.0 V), indicating a faster hole transport rate in PVK:C_8_–SS-devices. Subsequently, we performed electrochemical impedance spectroscopy (EIS) to investigate the kinetics of carrier transfer processes (Figure 4b). A simplified equivalent circuit model (inset in Figure 4b) was used to describe the processes occurring in the QLEDs, and the fitting parameters are shown in Table S3. The charge-transfer-related resistance (*R*_tr_) decreased from 5968 Ω (control device) to 3672 Ω (PVK:C_8_–SS device), indicating faster hole transport. The recombination resistance (*R*_rec_) decreased from 4765 Ω (control device) to 2960 Ω (PVK:C_8_–SS device), indicating a higher recombination rate. These results agree well with the *J–V* curves and demonstrate that the increasing performance in the PVK:C_8_–SS device originates from effective hole transport.

**Figure 4:**
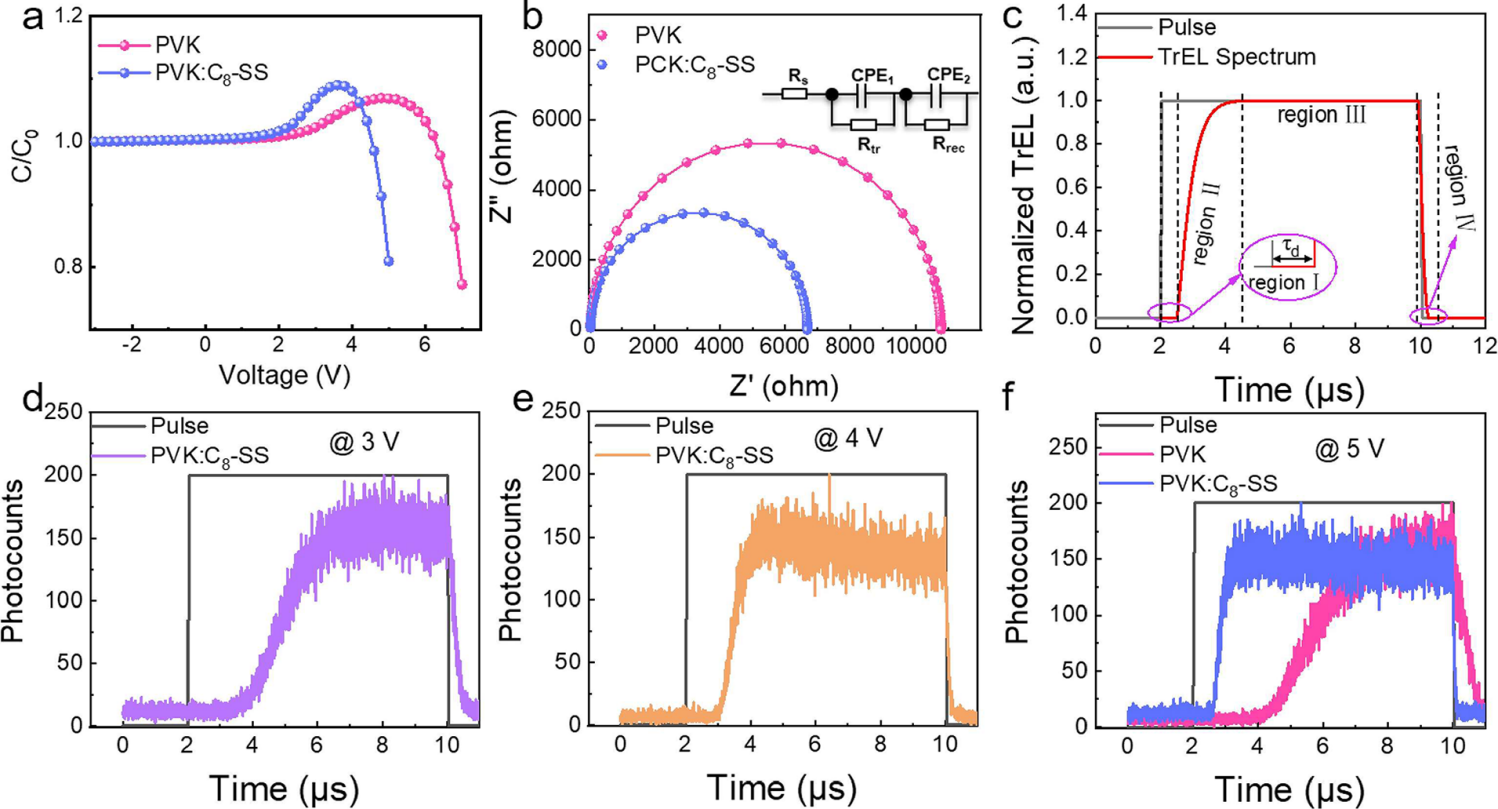
QLEDs’ (a) *C–V* characterization at 10 kHz and (b) Nyquist plots under 6.0 V. (inset) Model of an equivalent circuit. (c) Typical TrEL spectrum of the QLED. TrEL results of devices under biases of (d) 3, (e) 4, and (f) 5 V.

To further investigate the hole injection process in the QLED devices, we conducted TrEL measurements. Figure 4c presents a typical TrEL spectrum: the EL turn-on stage (region I), EL rise stage (region II), EL stable stage (region III), and EL decay stage (region IV). In region I, τ_d_ in region I represents the time delay between the onset of the periodic pulse signal and the EL signal. Since electrons transport faster than holes, τ_d_ can be interpreted as the time required for holes to traverse the HTL and inject into the QD.^45,46^Figure 4d–f depicts the TrEL spectra of the devices across a voltage range of 3.0–5.0 V. In PVK-based devices, TrEL signals could not be collected at biases of 3 and 4 until 5 V. However, in the PVK:C_8_–SS device, TrEL signals appeared at lower voltages, indicating a higher *V*_on_ for the control devices. Moreover, as the voltage increased, τ_d_ decreased. This behavior can be attributed to the significant influence of the voltage on hole mobility.

We analyzed the charge injection under different applied voltages to explain the improvement of device performance and the reduction of *V*_on_ (Figure 5).

**Figure 5:**
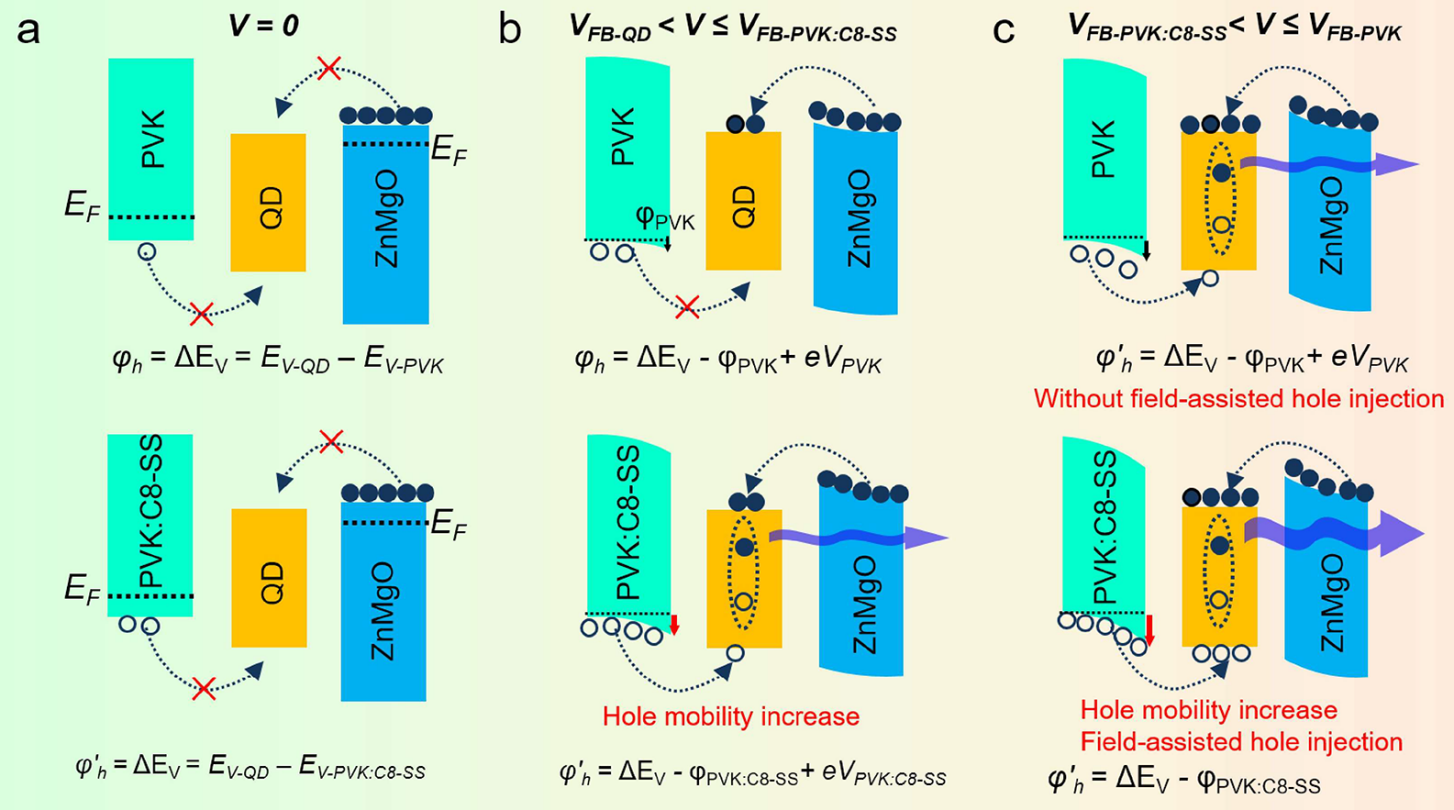
Summary of charge injection processes in QLEDs at different voltage.

(1) *V* = 0 V. Electrons and holes diffuse toward the region of lower concentration, creating a built-in field, also known as depletion region. The built-in field drifted the electrons and holes to the cathode and anode until the system reached thermal equilibrium. At this stage, both holes and electrons are unable to be injected into QDs for both devices. The hole injection barrier φ_h_ is
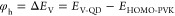
4where *E*_V-QD_ and *E*_HOMO-PVK_ are the valence band levels of QDs and the HOMO of PVK, respectively.

(2) *V*_FB-QD_ < *V* ≤ *V*_FB-PVK:C8-SS_. *V*_FB-QD_ and *V*_FB-PVK:C8-SS_ represent the flat-band voltages of the QDs and PVK:C_8_–SS, respectively. We use a *V*_FB-PVK:C8-SS_ reference point for analysis due to the narrower depletion region compared to PVK. The applied voltage, which is in the opposite direction to the built-in potentials, is mainly dropped across the depletion region. When the applied voltage increased to *V*_FB-QD_, electrons could be injected into QDs with a negligible barrier. However, for the PVK device, the hole injection barrier φ_h_ is
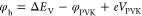
5for the PVK:C_8_–SS device, the hole injection barrier φ_h’_ is
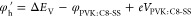
6where φ_PVK_ and φ_PVK:C8-SS_ are the downward energy band bending on PVK and PVK:C_8_–SS, respectively. φ_PVK:C8-SS_ is larger than φ_PVK_ due to the high hole concentration in PVK:C_8_–SS. *V*_PVK_ and *V*_PVK:C8-SS_ represent effective applied voltages dropped across the depletion region of PVK and PVK:C_8_–SS, respectively. *V*_PVK:C8-SS_ is smaller than *V*_PVK_ due to the narrower depletion region. Consequently, hole injection is possible in the PVK:C_8_–SS device, but it is not feasible in the PVK device. This leads to a lower *V*_on_ value for the PVK:C_8_–SS device.

(3) *V*_FB-PVK:C8-SS_ < *V* ≤ *V*_FB-PVK_. When the applied voltage is larger than that of *V*_FB-PVK:C8-SS_, the depletion region vanishes. The electric field in all layers turns positive, and the holes can be accelerated toward the QDs through field-assisted thermionic-emission mechanisms. However, in the PVK device, the existence of the depletion region still consumes part of the applied voltage, limiting hole injection. Therefore, the exciton recombination rate and efficiency in the PVK:C_8_–SS device are higher, resulting in better device performance.

## Conclusions

In summary, we successfully demonstrated an effective interfacial engineering strategy to develop efficient and stable blue QLEDs. The combination of PVK and C_8_–SS facilitates hole transport and charge balance due to the high hole mobility of C_8_–SS. The PVK:C_8_–SS-based blue QLEDs exhibited excellent performance with an EQE of 19.02% and a PE of 7.31 lm/W. Overall, our work demonstrates that treating HTLs with high hole mobility molecules is a simple and efficient way to develop high-performance blue QLEDs. Future research efforts should focus on designing and synthesizing thiopyran-based molecules or polymers with high mobility and electrochemical stability to further enhance the performance of blue QLEDs. Additional strategies such as incorporating polysulfide or selenium substitution have shown promise.^47^ Exploring electron-donating groups, such as amine groups or thiols, could provide valuable insights into optimizing the performance of optoelectronic devices.

## Supporting Information Available

Molecular structures of PVK and C_8_–SS; Calculated hole transfer integrals of C_8_–SS; PL spectra; TA response; UV–vis absorption spectrum of the QDs; Device performance of PVK with different amounts of C_8_–SS; CIE coordinate; UPS spectra; Optical transmission spectra; EQE statistics; Optical microscope images; Contact angles test; J-V characteristics of the EOD and HOD (PDF)
