# Supplementary material for: Charge Carrier Regulation for Efficient Blue Quantum-Dot Light-Emitting Diodes Via a High-Mobility Coplanar Cyclopentane[b]thiopyran Derivative
Source: Nano Lett. 2024 Apr 16;24(17):5284–91. doi: 10.1021/acs.nanolett.4c00883 (PMC11066960; doi:10.1021/acs.nanolett.4c00883)
Supplement: Supplementary file 1 — nl4c00883_si_001.pdf [file nl4c00883_si_001.pdf]

Supporting Information

Charge Carrier Regulation for Efficient Blue Quantum-Dot Light-Emitting Diodes via a High Mobility Coplanar Cyclopentane[*b*]thiopyran Derivative

Fensha Cai [†,1] Hao Zong,[‡,1] Meng Li,[†] Chenguang Li [†] Guangguang Huang,[†] Jorge Pascual,[§] Chao Liang,[ǁ] Zhenhuang Su,[▽] Zhe Li,[#] Xingyu Gao,[▽] Bo Hou,[⊥] Shujie Wang,[†] Gang Zhou,[*,‡] Zuliang Du[*,†]

[†] Key Lab for Special Functional Materials of Ministry of Education, National & Local Joint Engineering Research Center for High-efficiency Display and Lighting Technology, School of Materials Science and Engineering, and Collaborative Innovation Center of Nano Functional Materials and Applications,
Henan University,
Kaifeng 475004, China

[‡] Lab of Advanced Materials, State Key Laboratory of Molecular Engineering of Polymers, Fudan University, Shanghai 200438, P. R. China

[§] Polymat, University of the Basque Country UPV/EHU, 20018 Donostia-San Sebastian, Spain

[ǁ] MOE Key Laboratory for Nonequilibrium Synthesis and Modulation of Condensed Matter, School of Physics, Xi'an Jiaotong University, Xi'an 710049, P. R. China.

[▽] Shanghai Synchrotron Radiation Facility (SSRF), Shanghai Advanced Research Institute, Chinese Academy of Sciences, 239 Zhangheng Road, Shanghai 201204, China

[#] School of Engineering and Materials Science (SEMS), Queen Mary University of London, London, E1 4NS UK

[⊥] School of Physics and Astronomy, Cardiff University, Cardiff, Wales, CF24 3AA, UK

[1] These authors contributed equally to this work.
(G.Z.) Email: zhougang@fudan.edu.cn.
(Z.D.) Email: zld@henu.edu.cn.

Experimental Section

Materials. The electron transport layer of ZnMgO was synthesized according to our previous report.[1] Poly-*N*-vinylcarbazole (PVK, 99%), zinc acetate dihydrate ($Zn(OAc)_2$•$2H_2O$), magnesium acetate tetrahydrate ($Mg(OAc)_2$•$4H_2O$) were purchased from Sigma Aldrich. B-QDs were purchased from Suzhou Xingshuo Nanotech Co., Ltd. C_8-SS was synthesized as previously reported.[2]

Fabrication of QLEDs. The device structure was glass/ITO/PEDOT:PSS/HTLs/B-QDs/ZnMgO/Al. ITO substrates were ultrasonic with detergent (20 min at 45 °C), deionized water, acetone and isopropyl alcohol (15 min at room temperature) in sequence, then exposed to UV-ozone for 15 min. After UV-ozone treatment, PEDOT:PSS (AI 4083) solution was spin-coated on the ITO substrates at 5000 rpm for 30 s and baked at 130 °C for 15 min. All substrates were transferred to a N_2-filled glovebox. Subsequently, PVK (8.0 mg/mL in chlorobenzene) or PVK:C_8-SS hole transport layer was spin-coated on top of the PEDOT:PSS layer at 3000 rpm for 30 s and thermally annealed at 150 °C for 30 min. C_8-SS-modified PVK with the weight ratios of 1.2 , 3.6 , and 6.0 wt%. B-QDs dispersed in *n*-octane (18.0 mg/mL) and ZnMgO NPs were spin-coated at 3000 rpm for 30 s and baked at 60 °C for 30 min. Finally, thermally deposit Al cathode (100 nm) in a high-vacuum evaporation chamber with ~5×10^{-6} mbar. Then the devices are encapsulated with UV-resin and a cover glass. The effective area of the device is 0.04 cm^2.

Theoretical calculations. Gaussian 16 program was applied to make the theoretical calculations. The B3LYP method and 6-311G (d,p) basis set were used to conduct the

calculations of the molecular frontier orbitals. PW91 exchange and PW91 correlation functions with the 6-31G* basis set were used to calculate the transfer integrals between the HOMOs of adjacent molecules.[3, 4]

Characterization

The current density-voltage-luminance (*J-V-L*) characteristics of the blue QLEDs were carried out using a characterization system comprising a Keithley 2400 voltmeter together with a Photo Research 735 (PR-735) spectrometer under ambient conditions. X-ray photoelectron spectroscopy (XPS) measurement was measured with monochromatic Al Ka X-ray photons ($hv = 1486.6\ eV$) using an AXIS SUPRA+ System (Shimadzu Inc.). AFM morphology and c-AFM with a bias voltage of -2 V were performed by a SPA400 atomic force microscope. The UV-vis absorption was performed on a Lambda 950 PerkinElmer spectrometer. The grazing incidence X-ray diffraction (GIXRD) (BL14B1 beamline of the Shanghai Synchrotron Radiation Facility (SSRF) using X-ray with a wavelength of 1.24 Å) were used for characterizing the crystal characteristics of QDs film on PVK and PVK:C_8-SS HTLs. PL and TRPL spectra were performed on a HORIBA FluoroLog-3 spectrofluorometer, respectively. Helios pump-probe system (Ultrafast Systems LLC) coupled with an amplified femtosecond laser system (Coherent, 35 fs, 1 kHz, 800 nm) were used for measuring Femtosecond transient absorption (fs-TA) measurements. UPS spectra were acquired with a Thermo Scientific ESCALAB 250 XI equipment with a He I discharge lamp ($hv = 21.22\ eV$). The T_{50} lifetime of the devices was measured by a QLED life test system

of Newport Keithley N6705B. The capacitive voltage (*C-V*) curve was tested by Phystech FT 1030 deep level transient spectrometer. The electrochemical impedance spectra (EIS) were measured using Auto lab electrochemical workstation under dark conditions. The contact angle was measured by SL150L contact angle measuring instrument. TREL technology is a testing method that synchronously collects the electroluminescence (EL) response signal corresponding to the device by applying an electrical pulse signal. The TREL setup consists of a pulse generator (Keithley 4200A-SCS voltmeter, 50 MHz, rise/fall time at 10 ns, Sampling rate 200 MSa/s), Variable Gain Avalanche Detector (DeltaFlex from HORIBA), and time-correlated single-photon-counting (TCSPC). The supporting operating software is EzTime.

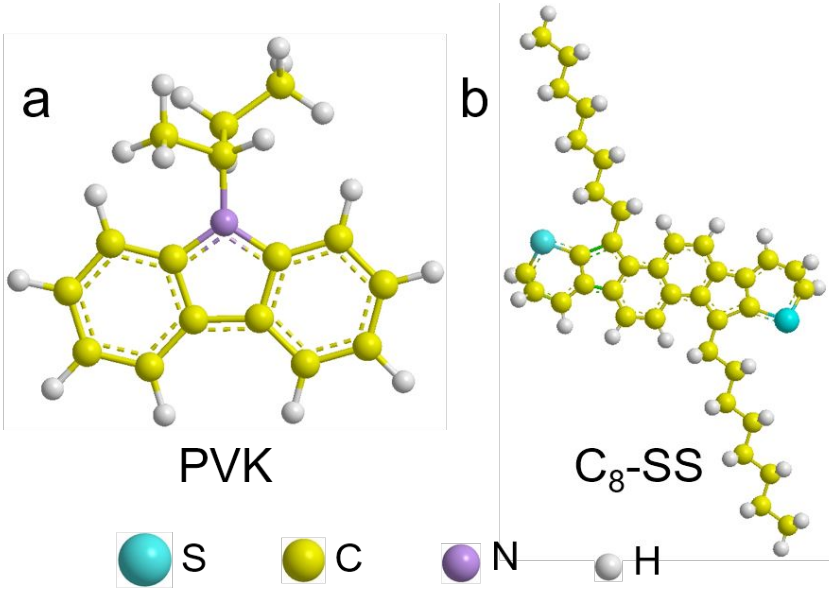

Figure S1. Molecular structures of (a) PVK and (b) C_8-SS.

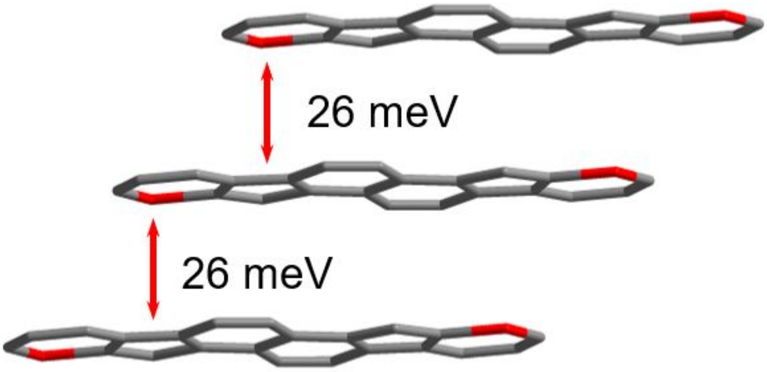

Figure S2. Calculated hole transfer integrals between the adjacent molecules of C_8-SS.

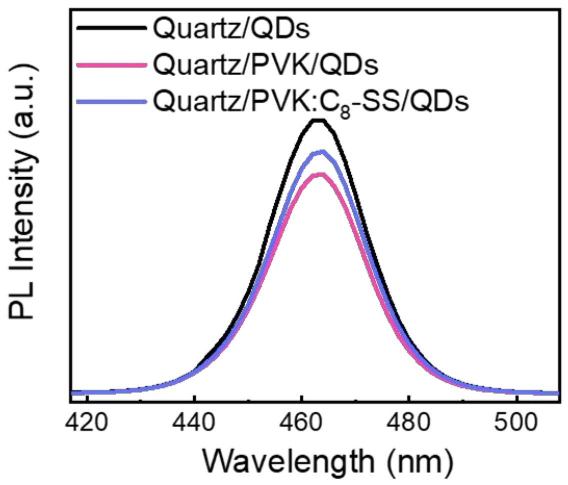

Figure S3. (a) PL spectra for the pristine QDs film, PVK/QDs film, and PVK:C_8-SS/QDs film deposited on quartz substrates.

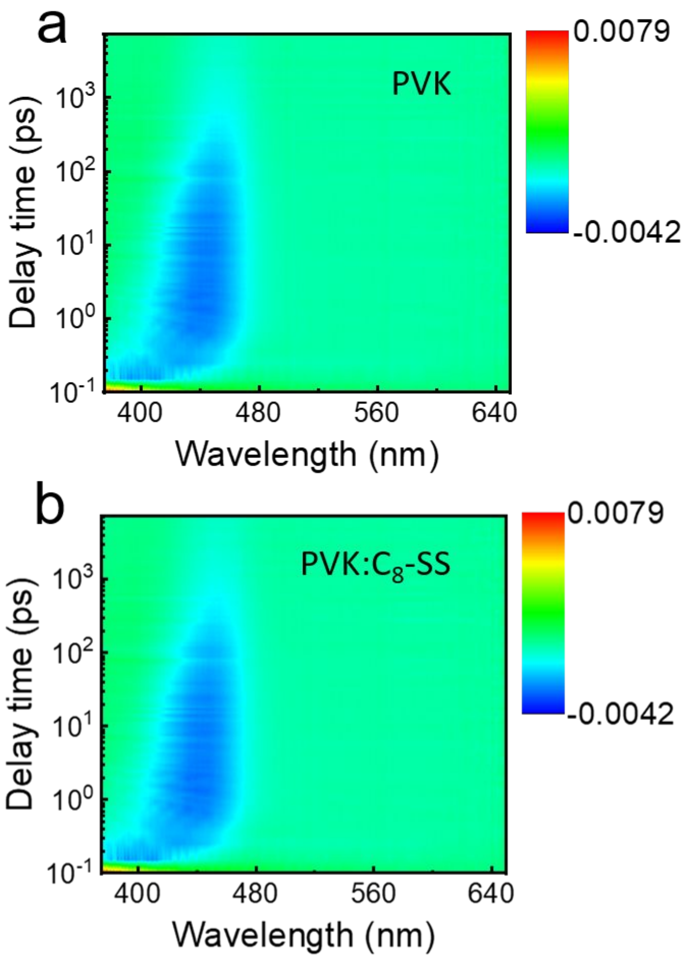

Figure S4. TAS response of (a) the PVK/QDs and (b) PVK:C_8-SS/QDs films deposited on quartz substrates.

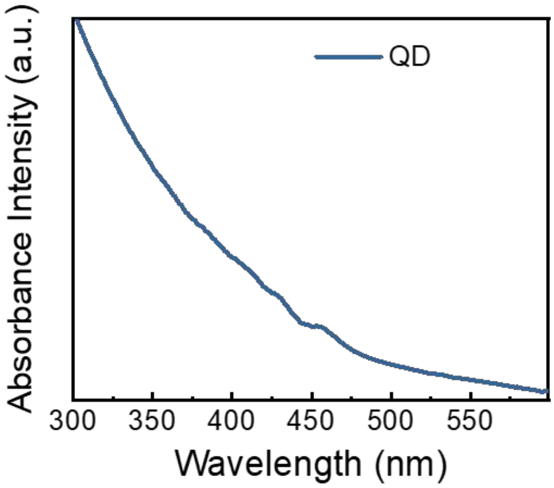

Figure S5. UV-vis absorption spectrum of the QDs.

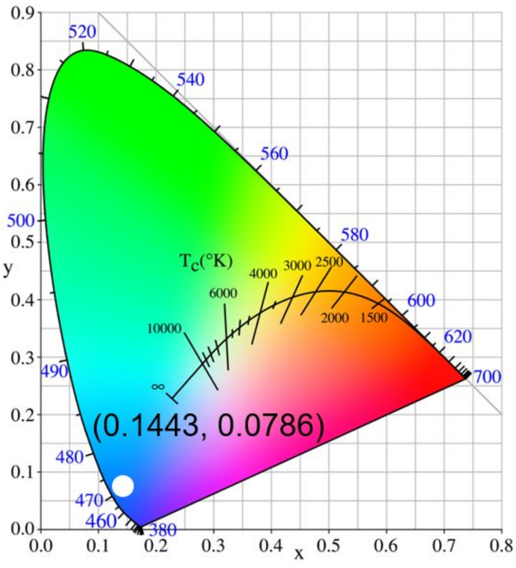

Figure S6. CIE coordinate of blue QLEDs.

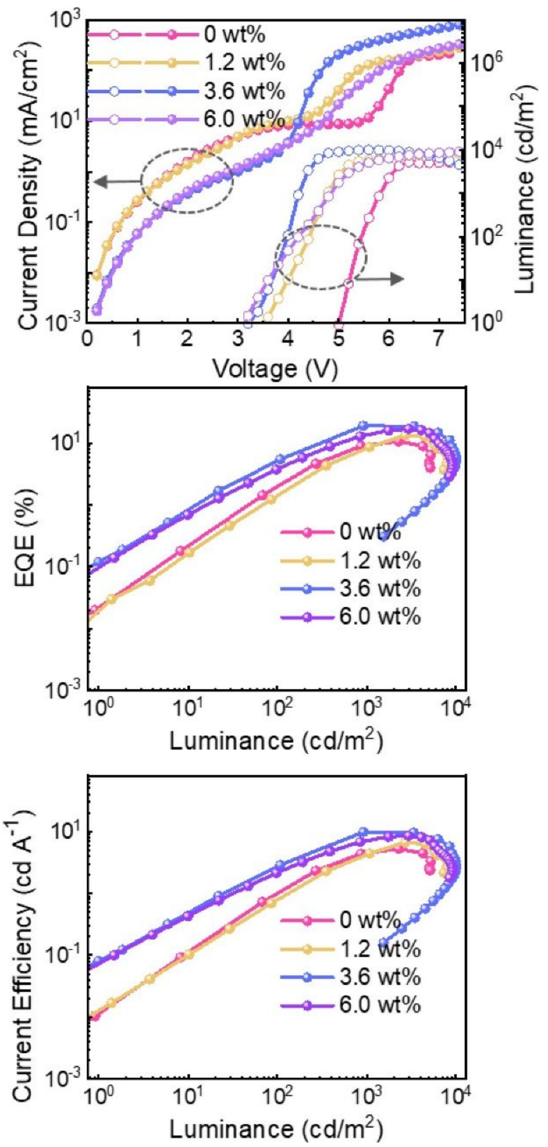

Figure S7. Device performance for PVK treated with different amounts of C_8-SS. (a) *J-V-L* characteristics. Luminance-dependent (b) EQE and (c) Current efficiency.

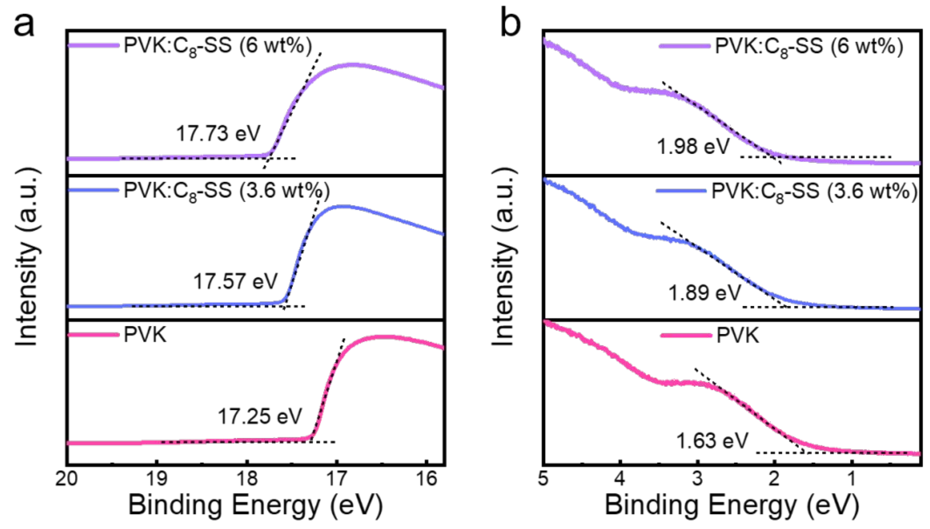

Figure S8. UPS spectra of PVK and PVK:C_8-SS films on ITO, showing (a) the secondary electron cut-off and (b) valence-band edge region.

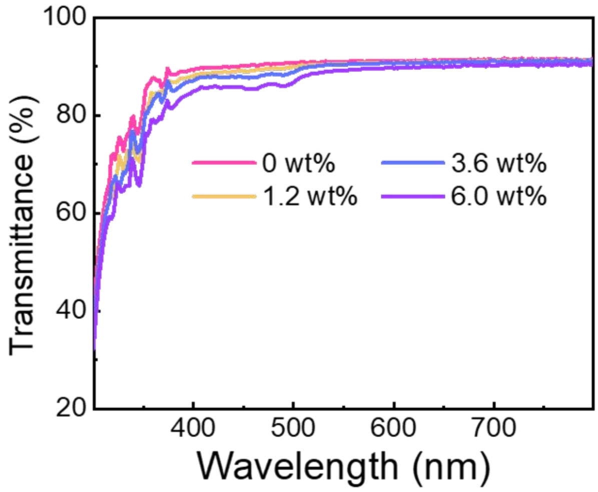

Figure S9. Optical transmission spectra of PVK films with different C_8-SS mass concentrations deposited on glass substrates.

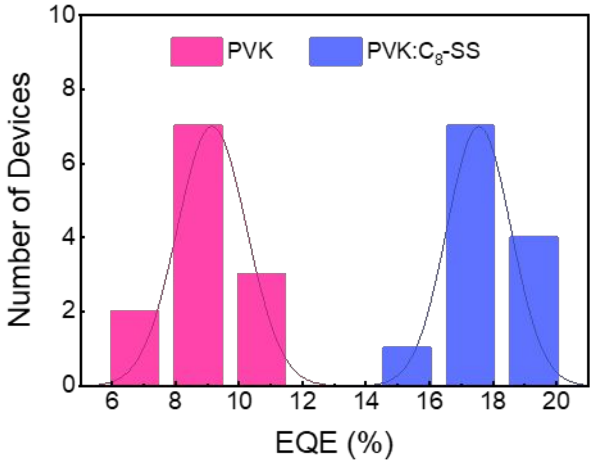

Figure S10. EQE statistics for QLEDs based on PVK and PVK:C_8-SS HTLs.

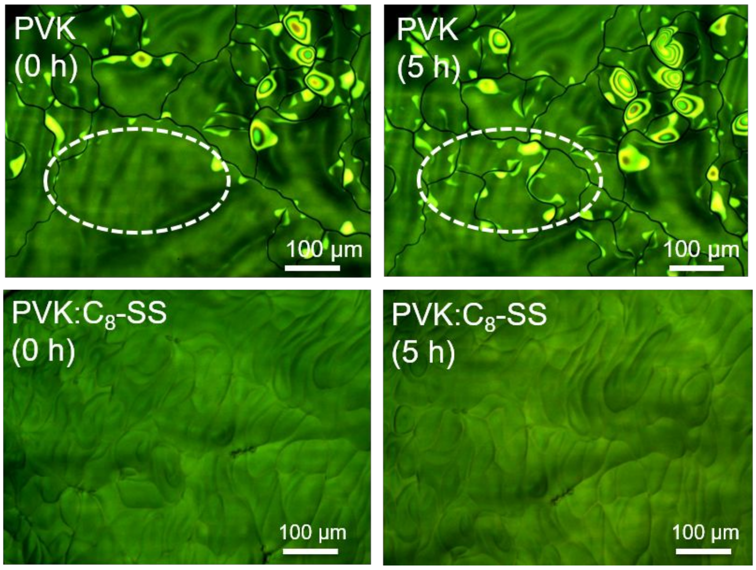

Figure S11. Optical microscope images of PVK and PVK:C_8-SS films on glass.

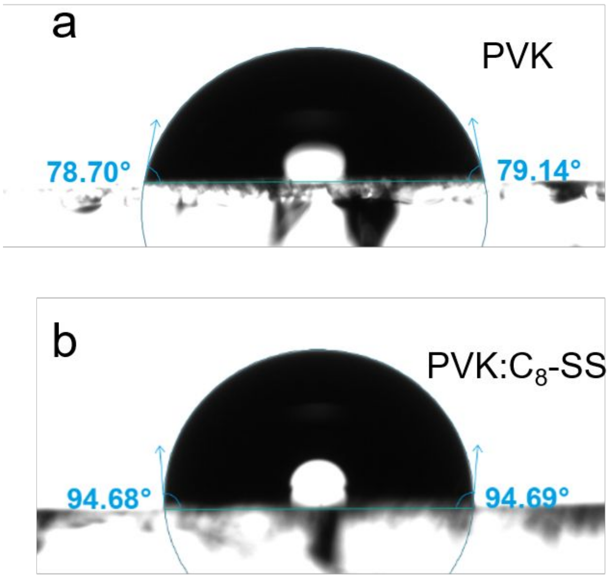

Figure S12. Water contact angles of (a) PVK and (b) PVK:C_8-SS HTLs.

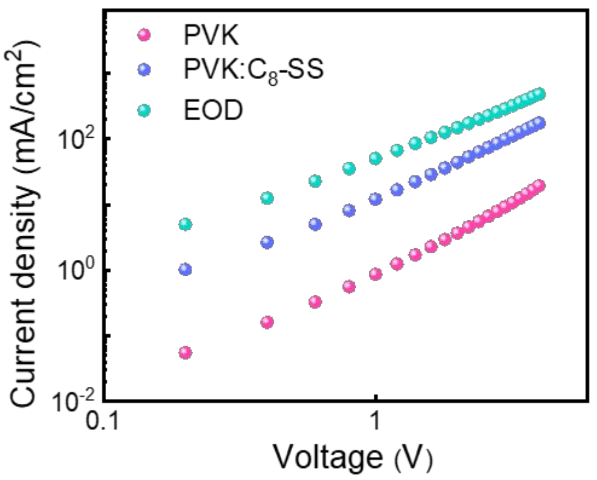

Figure S13. J-V characteristics of the EOD and HOD.

Table S1. Kinetic parameters obtained from TRPL spectra of Blue QDs deposited on Various HTLs.

Device	τ_1 (ns)	A_1 (%)	τ_2 (ns)	A_2 (%)	τ_{ave} (ns)	k_{CT} (10^7 s^{-1})	η_{CT} (%)
Quartz/QDs	0.88	24.66	8.52	75.34	6.63	/	/
Quartz/PVK/QDs	0.66	35.56	6.04	64.44	4.13	9.13	37.71
Quartz/PVK:C_8-SS/QDs	0.63	27.40	7.19	72.60	5.40	3.44	18.55

Table S2. Summary of Electroluminescence Performance of Blue QLEDs Based on PVK and PVK:C_8-SS HTLs.

Device	V_{on} (V)	L_{MAX} (cd/m^2)	CE (cd/A)	PE (lm/W)	EQE (%)
PVK	5.0	5216	5.27	2.76	10.71
PVK:C_8-SS (1.2 wt%)	3.6	8103	6.63	4.17	13.36
PVK:C_8-SS (3.6 wt%)	3.2	10023	9.77	7.31	19.02
PVK:C_8-SS (6.0 wt%)	3.2	9195	8.49	5.17	16.90

Table S3. Fitted impedance spectroscopy parameters for device based on PVK and PVK:C_8-SS HTLs.

Device	R_{rec} (Ω/cm^2)	R_{tr} (Ω/cm^2)	CPE1 (S·Secn/cm^2)	R_{rec} (Ω/cm^2)	CPE2 (S·Secn/cm^2)
PVK	40.39	5968	5.18*10^{-9}	4765	6.61*10^{-9}
PVK:C_8-SS	44.05	3672	4.58*10^{-9}	2960	5.59*10^{-9}

REFERENCES

(1) Cai, F.; Li, M.; Zhou, Y.; Tu, Y.; Liang, C.; Su, Z.; Gao, X.; Zeng, Z.; Hou, B.; Li, Z.; Aldamasy, M. H.; Jiang, X.; Wang, S.; Du, Z., Dipole-Tunable Interfacial Engineering Strategy for High-Performance All-Inorganic Red Quantum-Dot Light-Emitting Diodes. *Nano Energy* **2024**, 119, 109050.

(2) Qiao, Y.; Yang, L.; Zhu, J.; Yan, C.; Chang, D.; Zhang, N.; Zhou, G.; Zhao, Y.; Lu, X.; Liu, Y., Crystal Engineering of Angular-Shaped Heteroarenes Based on Cyclopenta[b]thiopyran for Controlling the Charge Carrier Mobility. *J. Am. Chem. Soc.* **2021,** *143* (29), 11088-11101.

(3) Xiaodi Yang, L. W., Caili Wang, Wei Long, and Zhigang Shuai, Influences of Crystal Structures and Molecular Sizes on the Charge Mobility of Organic Semiconductors: Oligothiophenes. *Chem. Mater.* **2008,** *20*, 3205–3211.

(4) Huang, J.; Kertesz, M., Intermolecular transfer integrals for organic molecular materials: can basis set convergence be achieved? *Chem. Phys. Lett.* **2004,** *390* (1-3), 110-115.
